# Supplementary material for: Novel Epoxides of Soloxolone Methyl: An Effect of the Formation of Oxirane Ring and Stereoisomerism on Cytotoxic Profile, Anti-Metastatic and Anti-Inflammatory Activities In Vitro and In Vivo
Source: Int J Mol Sci. 2022 Jun 1;23(11):6214. doi: 10.3390/ijms23116214 (PMC9181525; doi:10.3390/ijms23116214)
Supplement: Supplementary file 1 [file ijms-23-06214-s001.zip › ijms-1741323-supplementary.pdf]

Novel Epoxides of Soloxolone Methyl: an Effect of Formation of Oxirane Ring and Stereoisomerism on Cytotoxic Profile, Anti-metastatic and Anti-inflammatory Activities in vitro and in vivo

Oksana V. Salomatina [1,2,†], Aleksandra V. Sen'kova [1,†], Arseniy D. Moralev [1,3,†], Innokenty A. Savin [1], Nina I. Komarova [2], Nariman F. Salakhutdinov [2], Marina A. Zenkova [1] and Andrey V. Markov [1,*]

[1] Institute of Chemical Biology and Fundamental Medicine, Siberian Branch of the Russian Academy of Sciences, Lavrent'ev avenue, 8, 630090 Novosibirsk, Russia; ana@nioch.nsc.ru (O.V.S.) senkova_av@niboch.nsc.ru (A.V.S.); a.moralev@g.nsu.ru (A.D.M.); savin_ia@niboch.nsc.ru (I.A.S.); marzen@niboch.nsc.ru (M.A.Z.)
[2] N.N. Vorozhtsov Novosibirsk Institute of Organic Chemistry, Siberian Branch of the Russian Academy of Sciences, Lavrent'ev avenue, 9, 630090 Novosibirsk, Russia; komar@nioch.nsc.ru (N.I.K.); anvar@nioch.nsc.ru (N.F.S.)
[3] Novosibirsk State University, Pirogova Str., 1, 630090 Novosibirsk, Russia
* Correspondence: andmrkv@gmail.com; Tel.: +7-383-363-51-61 (A.V.M.)
† These authors contributed equally to this work.

NMR ^{1}H and ^{13}C, HRMS of βO-SM and αO-SM

Spectrum of **βO-SM,** ^{1}H NMR, 600MHz, $CDCl_3$

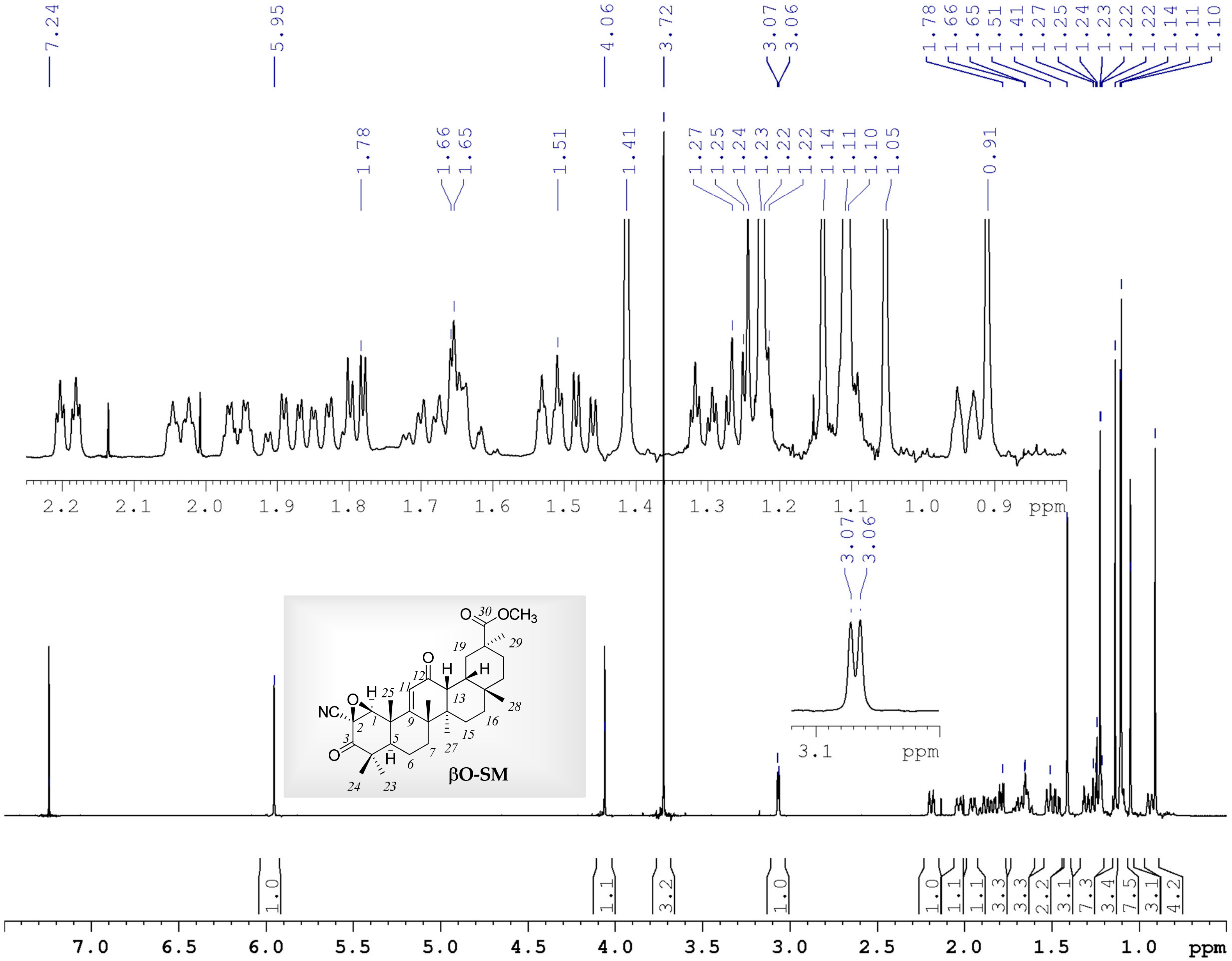

Spectrum of **βO-SM,** ^{13}C NMR JMOD, 150MHz, $CDCl_3$

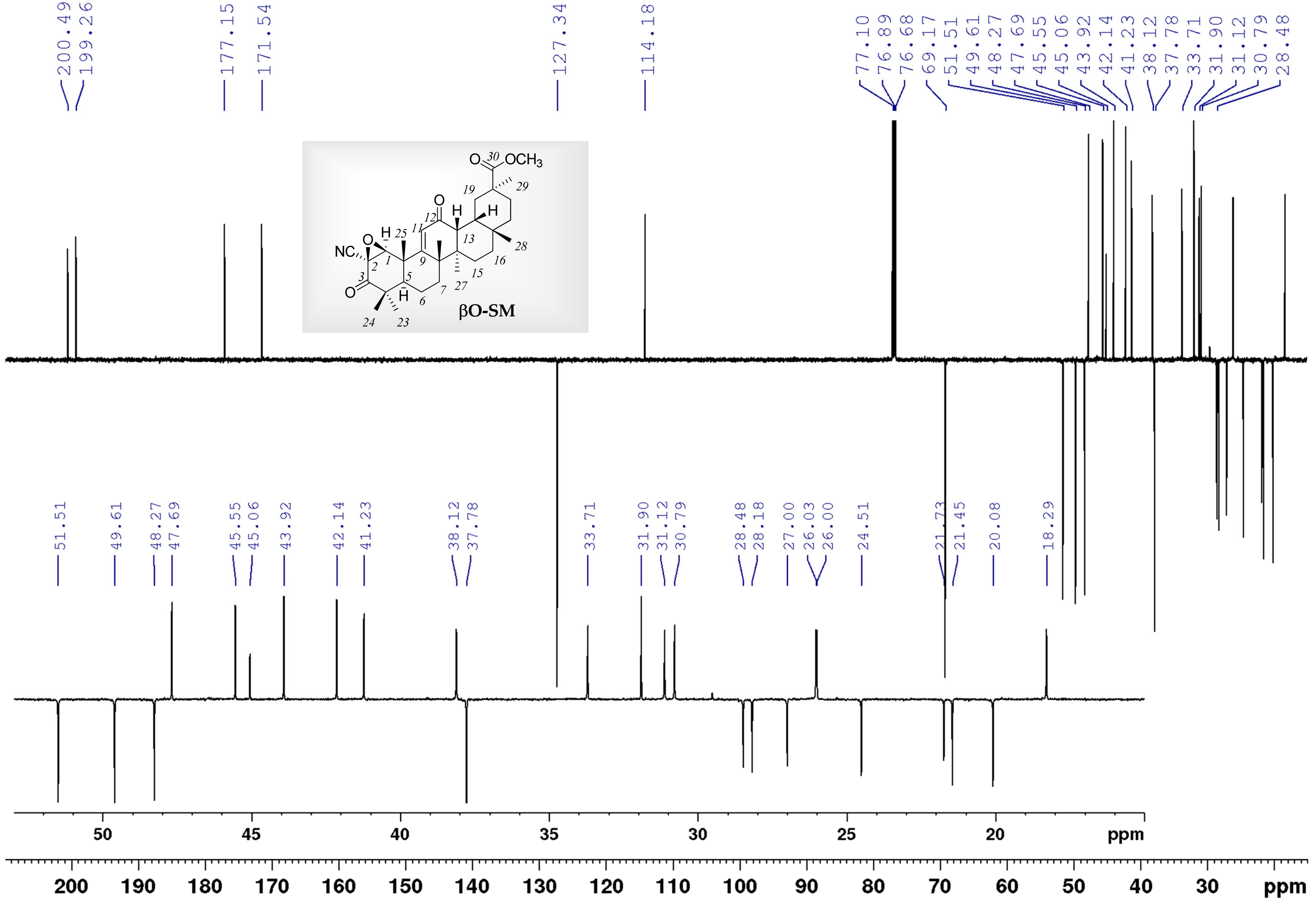

Spectrum of **βO-SM,** ^{1}H-^{1}H NOESY NMR, 600MHz, $CDCl_3$

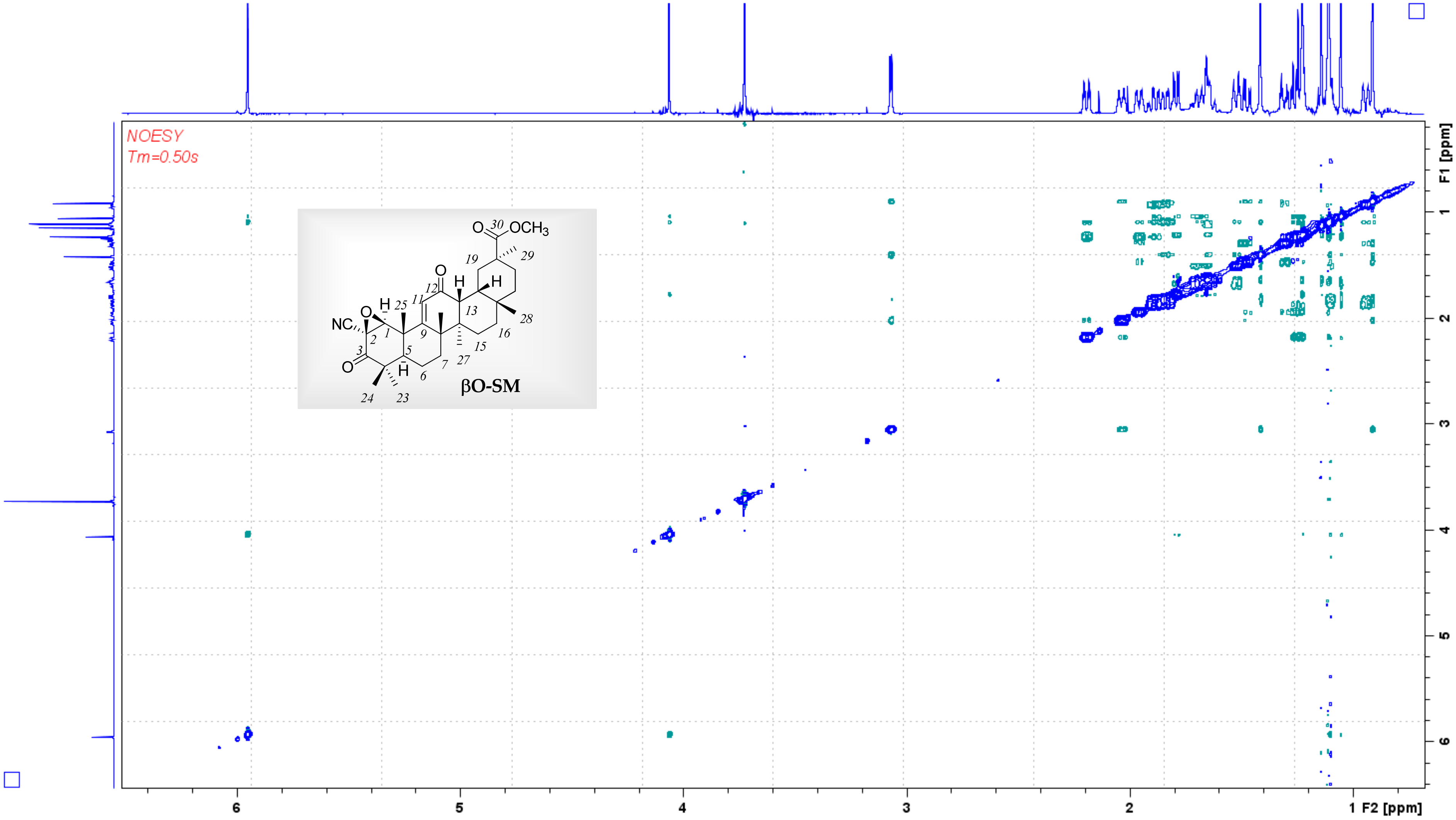

High resolution mass spectrum of **βO-SM**, Tsource=120°C, Tprobe=280°C

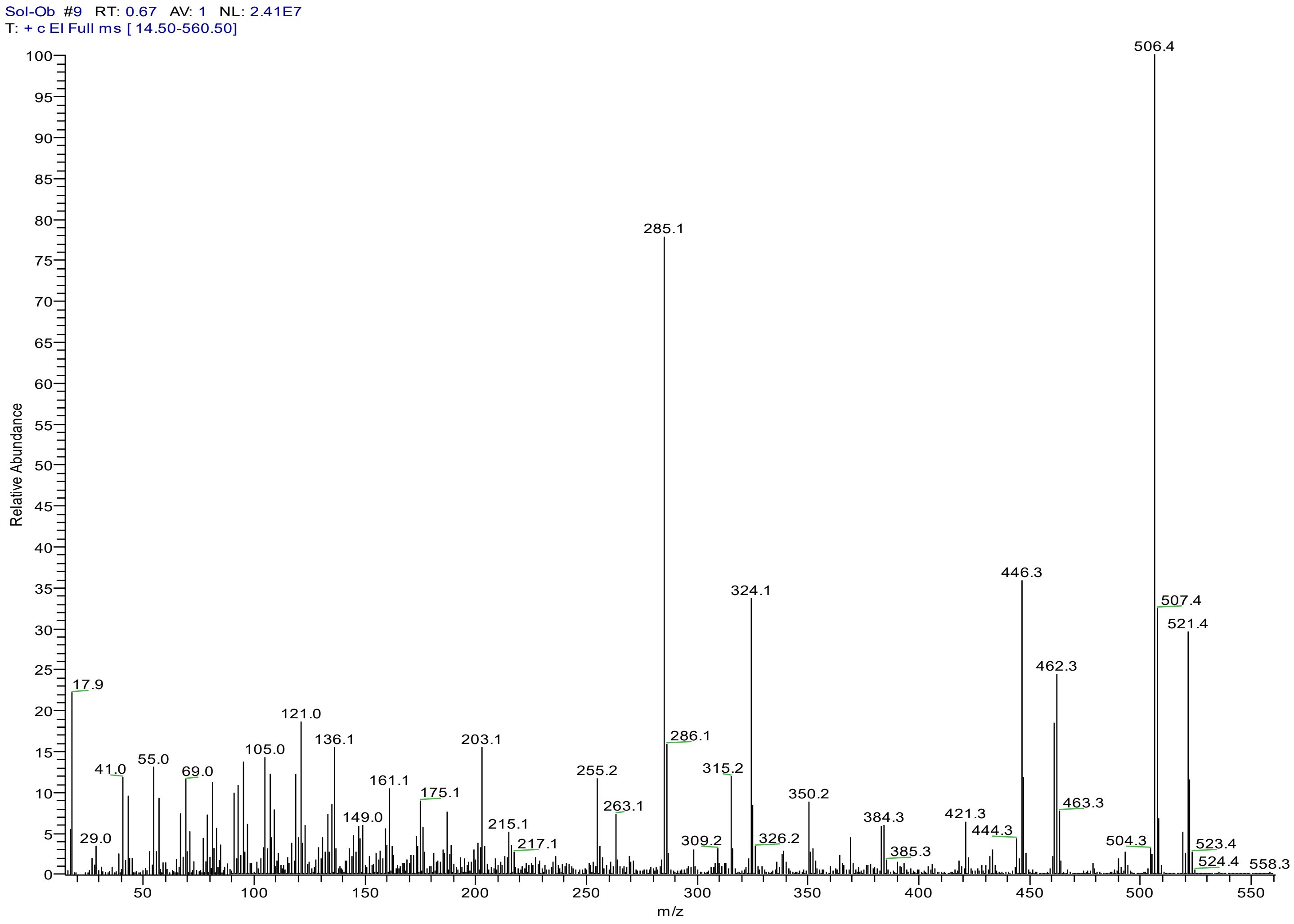

Calculated m/z= 521.3136 $(C_{32}H_{43}O_5N_1)^+$

Found m/z= 521.3131

Spectrum of **αO-SM,** ^{1}H NMR, 600MHz, $CDCl_3$

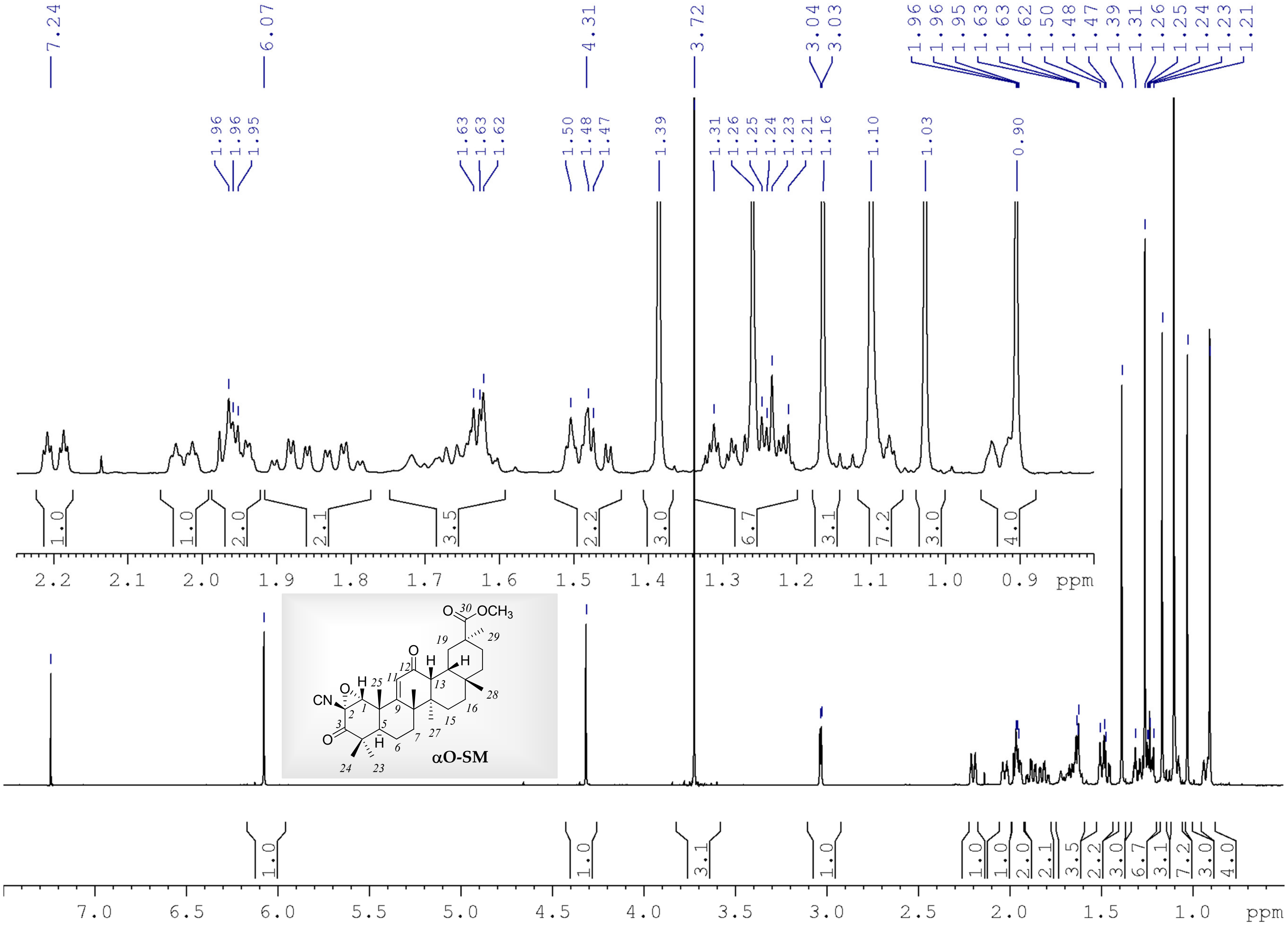

Spectrum of **αO-SM,** ^{13}C NMR JMOD, 150MHz, $CDCl_3$

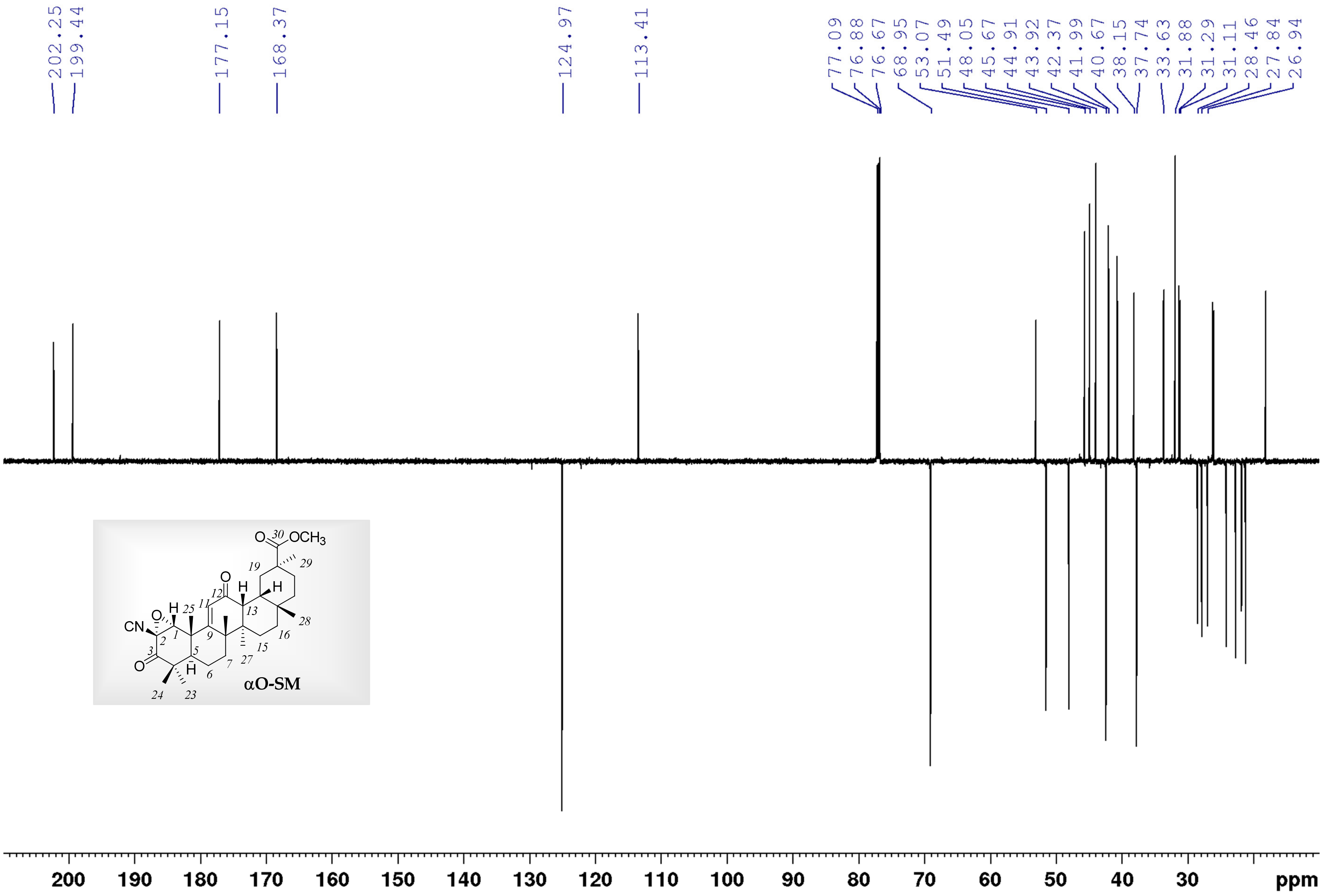

Spectrum of **αO-SM,** ^{1}H-^{1}H NOESY NMR, 600MHz, $CDCl_3$

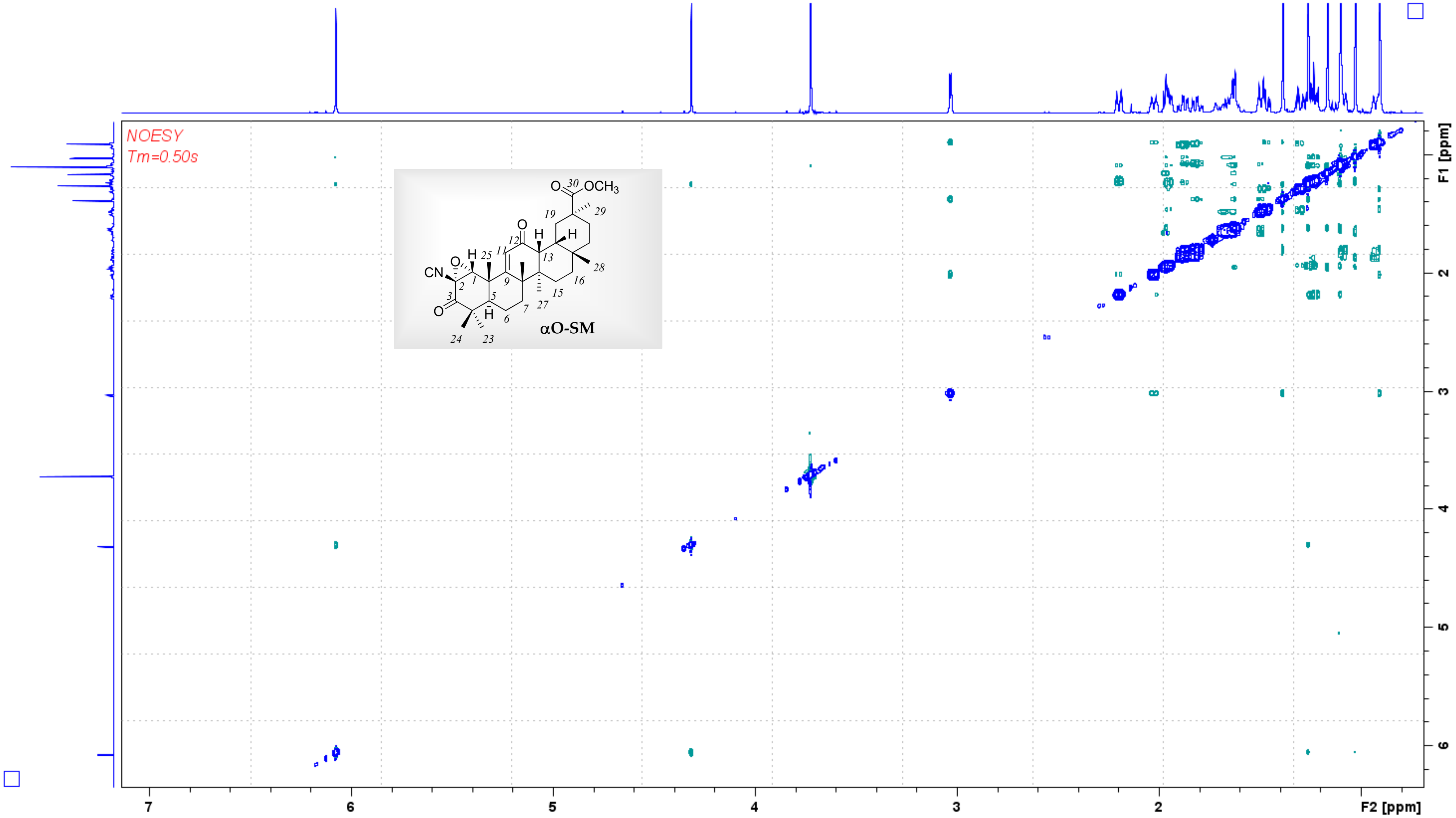

High resolution mass spectrum of **αO-SM**, Tsource=110°C, Tprobe=250°C

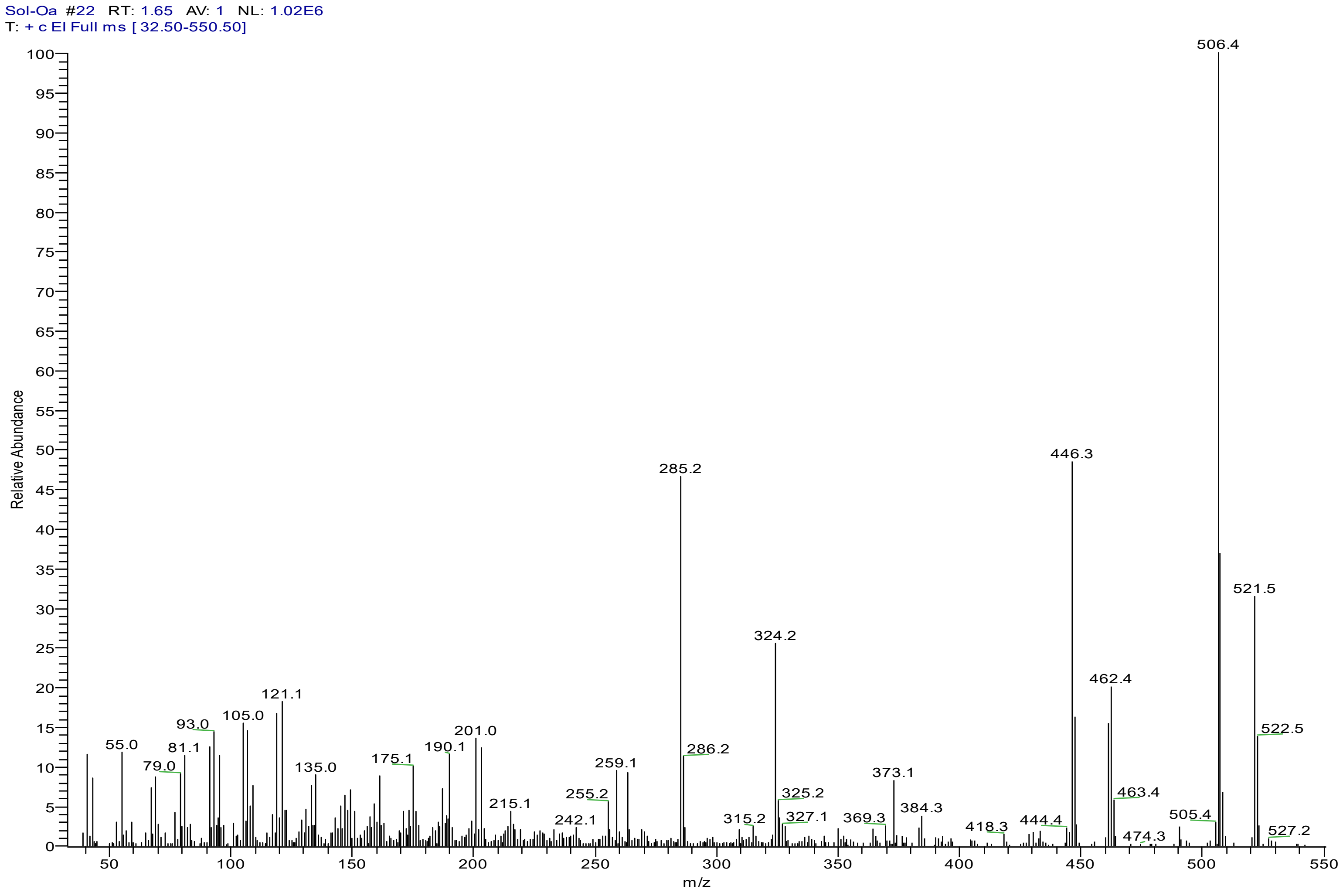

Calculated m/z= 521.3136 $(C_{32}H_{43}O_5N_1)^+$

Found m/z= 521.3131
